# Identification and treatment of intestinal malrotation with midgut volvulus in childhood: a multicenter retrospective study

**DOI:** 10.3389/fped.2024.1390856

**Published:** 2024-05-13

**Authors:** Xiaofeng Yang, Wei Wang, Kun Wang, Jingquan Zhao, Liandong Sun, Shuai Jiang, Yewen Wang, Wenyu Feng, Guojian Ding, Tingliang Fu, Aiwu Li, Lei Geng

**Affiliations:** ^1^ Department of Pediatric Surgery, Binzhou Medical University Hospital, Binzhou, Shandong, China; ^2^ Department of Surgery, Maternity and Child Health Care of Zaozhuang, Zaozhuang, Shandong, China; ^3^ Department of Pediatric Surgery, The People’s Hospital of Rizhao, Rizhao, Shandong, China; ^4^ Department of Surgery, Zibo Maternal and Child Health Hospital, Zibo, Shandong, China; ^5^ Department of Pediatric Surgery, Liaocheng People’s Hospital, Liancheng, Shandong, China; ^6^ Department of Pediatric Surgery, Qilu Hospital of Shandong University, Jinan, Shandong, China

**Keywords:** intestinal malrotation, midgut volvulus, Ladd’s procedure, bilious vomiting, children

## Abstract

**Background:**

Intestinal malrotation is a rare condition, and its delayed diagnosis can lead to fatal consequences. This study aimed to investigate the identification and treatment of malrotation in children.

**Methods:**

Clinical data, imaging, operative findings, and early postoperative outcomes of 75 children with malrotation were retrospectively analyzed.

**Results:**

The mean age was 6.18 ± 4.93 days and 51.26 ± 70.13 months in the neonatal group (56 patients) and non-neonatal group (19 patients), respectively. Sixty-seven patients were under the age of 1 year at the time of diagnosis. The occurrence of bilious vomiting and jaundice was significantly higher in the neonatal group (89.29%) than that in the non-neonatal group (37.5%), *p* < 0.05 and *p* < 0.01, respectively. The incidence of abnormal ultrasound (US) findings was 97.30% and 100%, respectively, and the sensitivities of the upper gastrointestinal series were 84.21% and 87.5%, respectively. Sixty-six (88%) patients had midgut volvulus, including *in utero* volvulus (two patients) and irreversible intestinal ischemia (four patients). Most neonates (89.29%) underwent open Ladd's procedure with a shorter operative time (*p* < 0.01). Reoperation was performed for postoperative complications (four patients) or missed comorbidities (two patients).

**Conclusions:**

Non-bilious vomiting was the initial symptom in >10% of neonates and nearly 40% of non-neonates. This highlights the importance for emergency physicians and surgeons to be cautious about ruling out malrotation in patients with non-bilious vomiting. Utilizing US can obviate the need for contrast examinations owing to its higher diagnostic accuracy and rapid diagnosis and can be recommended as a first-line imaging technique. Additionally, open surgery is still an option for neonatal patients.

## Introduction

Intestinal malrotation, a congenital rotational anomaly, refers to the abnormalities of the intestinal position resulting from non-rotation, incomplete rotation, or abnormal fixation of the embryonic gut (1). The distal duodenum does not reach the left side of the abdomen and, therefore, lacks the stability usually provided by the ligament of Treitz. It is estimated that 3.9–20 of 10,000 newborns present with intestinal malrotation (2–4), which may complicate acute, intermittent, or chronic midgut volvulus in children and adults (5). A high index of suspicion is vital for its accurate and rapid identification (5). However, acute malrotation with volvulus, a life-threatening emergency with high morbidity and mortality, is difficult to diagnose in the non-verbal pediatric populations (3, 6, 7).

Upper gastrointestinal series (UGIs) remains the gold standard for diagnosing malrotation with or without volvulus (8). However, in recent years, ultrasound (US) has been used as the first-line modality for diagnosing malrotation with volvulus (9), even during prenatal screening (10, 11). The treatment of malrotation with volvulus is challenging for emergency physicians and pediatric surgeons. Ladd's procedure is curative for malrotation with good outcomes; however, a higher rate of emergency reoperation has been reported in older children (12). Moreover, controversy remains regarding a surgical approach to Ladd's procedure, and there is no evidence that a laparoscopic Ladd's procedure is superior to open surgery, especially during the neonatal period (5, 13–17).

This study aimed to review the multicenter experience to provide useful information regarding the identification of intestinal malrotation and evidence to support the superiority of laparoscopic Ladd's procedure over open surgery or vice versa in pediatric patients.

## Patients and methods

### Clinical data

Clinical data from six tertiary hospitals between January 2006 and June 2022 were retrospectively reviewed. The inclusion criteria were those aged ≤18 years and had surgically confirmed intestinal malrotation. Keywords including intestinal malrotation, congenital intestinal malrotation, volvulus, duodenal stenosis or atresia, and annular pancreas were used in the digital integrated case management system. The variables, including demographics (sex, age, and birth weight), medical history, symptoms and signs, laboratory and radiological findings, surgical procedures, and outcomes (operative time, time to initial enteral feeding, length of hospital stay, and occurrence of complications) were collected and analyzed. The time to diagnosis was defined as the period from admission to the initiation of anesthesia. Based on the age at admission, the patients were divided into two groups: the neonatal group (aged ≤28 days) and the non-neonatal group (aged >28 days and ≤18 years). Based on the different surgical approaches, the patients were divided into three groups: open, laparoscopic, and laparoscopic conversion to open Ladd's procedure.

### Ethical considerations

All procedures performed in the study involving human participants were in accordance with the ethical standards of the institutional and/or National Research Committee, and the present study adhered to and was conducted according to the principles of the Declaration of Helsinki in 1964 and its subsequent amendments or comparable ethical standards. Institutional review board approval was not needed, as it is a retrospective analysis. Written informed consent was obtained from the parents/legal guardians of all children involved in the study.

### Statistical analysis

All values of quantitative and qualitative variables were expressed as mean and standard deviation or as a percentage. Comparisons were made using a *t*-test or *χ*^2^ test. SPSS software (version 20.0; SPSS, Inc., Chicago, IL, USA) was used for the statistical analysis. A *p*-value of <0.05 was considered statistically significant.

## Results

Seventy-five patients met the inclusion criteria. The data for the entire cohort of patients based on age of onset are summarized in Table 1. Of the 75 patients, 56 (74.67%) belong to the neonatal group and 19 (25.33%) belong to the non-neonatal group. The mean age was 6.18 ± 4.93 days and 51.26 ± 70.13 months in the neonatal and non-neonatal groups, respectively. Approximately three-quarters of patients (76%) were male. Patients presented with vomiting (98.67%), bilious vomiting (82.67%), abdominal distension (37.33%), jaundice (29.33%), hematochezia (9.33%), and hematemesis (2.67%). Vomiting occurred in all neonates and in 94.74% of the non-neonatal patients. Of the 56 neonatal patients, 50 presented with bilious emesis and 21 had jaundice, while in the non-neonatal group, 12 presented with bile-stained emesis and 1 with jaundice (*p* = 0.024 and *p* = 0.008, respectively).

**Table 1:** Comparison of clinical data of the patients.

Variable	Total	Newborns	Non-neonatal cases	*p*-value
Number of patients (*n*, %)	75	56 (74.67)	19 (25.33)	
Gender (male:female)	57:18	44:12	13:6	0.559
Mean age (days), mean (SD)		6.18 (4.93)	51.26 (70.13)	
Mean weight (kg), mean (SD)		3.05 (0.58)	19.02 (19.89)	
Initial clinical manifestation				
Emesis (*n*, %)	74 (98.67)	56 (100)	18 (94.73)	0.253
Bilious emesis (*n*, %)	62 (82.67)	50 (89.29)	12 (63.16)	0.024
Non-bilious emesis (*n*, %)	12 (16.00)	6 (10.71)	6 (31.58)	
Hematemesis (*n*, %)	2 (2.67)	1 (1.79)	1 (5.26)	0.445
Hematochezia (*n*, %)	7 (9.33)	7 (12.50)	0	0.117
Jaundice (*n*, %)	22 (29.33)	21 (37.50)	1 (5.26)	0.008
Abdominal distension (*n*, %)	28 (37.33)	22 (39.29)	6 (31.58)	0.548
Abdominal x-rays (*n*, %)	39	32 (57.14)	7 (36.84)	0.126
Abnormal findings	30	25	5	0.653
Double bubble sign	4	4	0	0.462
Air–fluid level	20	15	5	0.109
Intestinal gas accumulation	4	4	0	0.462
Pneumoperitoneum	1	1	0	0.833
Intestinal obstruction	1	1	0	0.833
UGIs (*n*, %)	27	19 (33.93)	8 (42.11)	0.521
Duodenal obstruction/DJJ abnormal position (*n*, %)	23	16 (84.21)	7 (87.50)	0.663
Contrast enema (*n*, %)	13	9 (16.07)	4 (21.05)	0.885
Abnormal position of the cecum (*n*, %)	12	8 (88.89)	4 (100)	0.692
US (*n*, %)	48	37 (66.07)	11 (57.89)	0.521
Abnormal findings	47	36 (97.30)	11 (100)	0.771
Ascites	6	4	2	
Duodenal dilatation	20	17	3	0.411
SMA-SMV inversion	35	27	8	1.000
“whirlpool” sign	24	21	3	0.071
Intestinal wall thickening	1	0	1	0.234
CT scan (*n*, %)	2	0	2 (10.53)	0.062
“whirlpool” sign	2	0	2	
Time from onset to diagnosis [median time (hours)]		22.5 (2.5–480)	17 (2–288)	
≤12 h (*n*, %)		20 (35.71)	7 (36.84)	0.916
>12–24 h (*n*, %)		7 (12.5)	3 (15.79)	
≥24 h (*n*, %)		29 (51.79)	9 (47.37)	
Intraoperative findings				
Without volvulus (*n*, %)	9	8 (14.29)	1 (5.26)	0.524
With volvulus (°) (*n*, %)	66	48 (85.71)	18 (94.74)	0.876
180	14	9	5	
270	7	5	2	
360	26	20	6	
540	7	6	1	
720	12	8	4	
Irreversible bowel ischemia	5	4	1	
Chylous ascites/cyst (*n*, %)	12	7	5	0.290
Comorbid congenital anomalies (*n*, %)	20	15 (26.79)	5 (26.32)	0.968
Annular pancreas		4 (26.67)	0	
With congenital cardiac disease		1	0	
With Down's syndrome		1	0	
Duodenal web		3 (20.00)	0	
With Meckel diverticulum		1	0	
Intestinal atresia		3 (20.00)	0	
Heterotopic pancreas		2 (13.33)	1	
Intestinal duplication malformation		0	1	
Umbilical hernia		0	1	
Hydronephrosis		0	1	
Cyst of the mesentery		0	1	
Surgical approach				0.000
Open Ladd's (*n*, %)	59	50 (89.29)	9 (47.37)	
Laparoscopic Ladd's (*n*, %)	6	1 (1.79)	5 (26.32)	
Laparoscopic conversion to open Ladd's (*n*, %)	10	5 (8.93)	5 (26.31)	

SMV, superior mesenteric vein; SMA, superior mesenteric artery; UGIs, upper gastrointestinal series; US, ultrasound; CT, computed tomography; DJJ, duodenal–jejunal junction.

The median time to confirm malrotation was 22.5 h (2.5–480 h) in the neonatal group and 17 h (2–288 h) in the non-neonatal group (*p* = 0.916). More than 24 h was required to confirm the diagnosis in half of the patients (50.67%).

**Figure 1:**
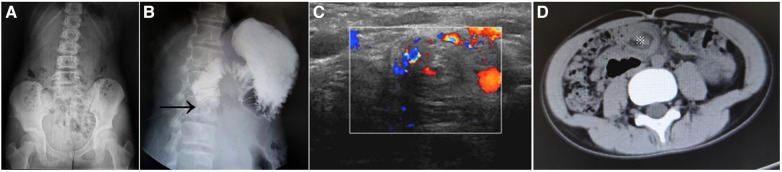
Plain abdominal film showed scant intestinal gas (**A**). The UGIs demonstrate obstruction of the second part of the duodenum (arrow) (**B**). Ultrasound shows the “whirlpool” sign, suggestive of malrotation with volvulus of 270°−360° (**C**). Axial CT image shows the “whirlpool” sign (asterisk) (**D**). UGIs, upper gastrointestinal series; CT, computed tomography.

The diagnostic tools included US in 48, abdominal radiography in 39, UGIs in 27, and contrast enema in 13. Abnormal plain abdominal radiographs were observed in 30 patients, including air–fluid level, double bubble sign, intestinal gas accumulation, intestinal obstruction, and pneumoperitoneum. Air–fluid level or scant intestinal gas (Figure 1A) was found in five non-neonatal patients. UGIs revealed duodenal obstruction and/or an abnormal duodenal position of the duodenojejunal junction in 16/19 (84.21%) in the neonatal group and 7/8 (87.50%) in the non-neonatal group (Figure 1B). The contrast enema showed an abnormal position of the cecum in eight out of nine (88.89%) neonatal patients and in all four non-neonatal patients.

Abnormal US findings were found in most neonatal patients (36/37, 97.3%) and all 11 (100%) non-neonatal patients, including superior mesenteric artery–superior mesenteric vein (SMA-SMV) inversion (*n* = 27 vs. *n* = 8), “whirlpool” sign (Figure 1C) (*n* = 21 vs. *n* = 3), duodenal dilation (*n* = 17 vs. *n* = 3), and ascites (*n* = 4 vs. *n* = 2) between the neonatal and non-neonatal groups. Two non-neonatal patients showed a “whirlpool” sign at the base of the small bowel mesentery via CT scan (Figure 1D).

Open Ladd's procedure was performed in 50/56 (89.29%) of neonatal patients and 9/19 (47.37%) of non-neonatal patients, the laparoscopic procedure was performed in 1/56 (1.79%) and 5/19 (26.32%), and the remaining 10 patients required laparoscopic conversion to an open Ladd's procedure in 5/56 (8.93%) and 5/19 (26.32%), respectively. Operative time, initial postoperative feeding time, and hospital stay for the three surgical approaches are shown in Table 2. The operative duration for laparoscopic conversion to open Ladd's group (224.10 ± 61.234 min) differed significantly from that of open Ladd's (122.76 ± 65.429 min) and laparoscopic (134.00 ± 35.07 min) procedures (*p* < 0.01). However, no significant differences were detected between the open and laparoscopic Ladd's procedure groups. Similarly, there were no significant differences in the initial postoperative oral feeding time and length of hospital stay among the three surgical approach groups.

**Table 2:** Operative time and postoperative outcome among the three groups.

Variables	Open Ladd's (*n* = 59)	Laparoscopic Ladd's (*n* = 6)	Laparoscopic conversion to open Ladd's (*n* = 10)	*p*-value (intergroup comparisons)
Operative time (minutes), mean (SD)	122.76 (65.429)	134.00 (35.07)	224.10 (61.234)	<0.01
Time to initial oral feeding (days), mean (SD)	4.47 (2.381)	4.50 (4.722)	3.40 (1.578)	0.221
Hospital stay (days), mean (SD)	14.51 (7.302)	14.17 (5.115)	14.40 (5.700)	0.981
In-hospital mortality, *n*	0	0	0	
30-day mortality, *n*	0	0	0	

**Figure 2:**
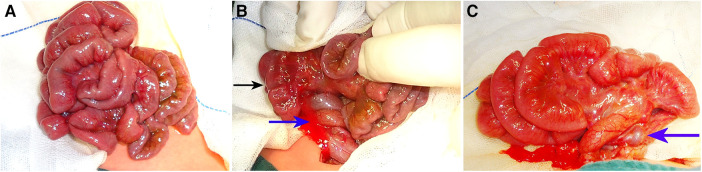
An 8-day-old boy with bilious vomiting for 8 h, malrotation with midgut volvulus suspected by UGIs. Intraoperative views by open surgical approach show complete small bowel ischemia (black arrow) (**A**,**B**) due to a midgut torsion of 720° (blue arrow) (**B**). After torsion reduction, intestinal viability is restored (**C**), and the dilated mesenteric vein caused by venous congestion is shown (violet arrow). UGIs, upper gastrointestinal series.

Intraoperative findings revealed 66 patients of complicated midgut volvulus (Figures 2A–C), including 48 neonates (180° in 9 patients, 270° in 5 patients, 360° in 20 patients, 540° in 6 patients, and 720° in 8 patients). Intestinal torsion of 360° was observed in one patient and 720° in two patients, which caused irreversible intestinal ischemia of 30, 35, and 80 cm in length, respectively, and required intestinal resection and primary anastomosis. Eighteen non-neonatal patients were complicated by midgut volvulus with torsions of 180° (five patients), 270° (two patients), 360° (six patients), 540° (one patient), and 720° (four patients).

Five neonates required reoperation because of early postoperative recurrent bowel volvulus (one patient), adhesive bowel obstruction (two patients), iatrogenic defect of the hepatic falciform ligament that caused internal hernia and acquired jejunal atresia (one patient), or missed coexisting annular pancreas (one patient). Postoperative recovery was uneventful in all patients.

## Discussion

The midgut, perfused by the superior mesentery artery, completes its normal rotation and fixation at 10 weeks of gestation. Malrotations occur in approximately 0.2%–1% of the population (4). Abnormal gut rotation and fixation to the retroperitoneum/peritoneum lead to malrotation of the intestine, usually including non-rotation, malrotation or incomplete rotation, and inverse rotation, depending on the degree and direction of midgut rotation (18, 19). In our case series, all patients had malrotation or incomplete malrotation, which was rotated by 180° and recessed. Abnormal midgut fixation leads to a short mesenteric attachment, which makes the midgut prone to twisting (volvulus) around the SMA and SMV (4, 20–24).

Williams et al. (20) reported that the incidence of malrotation with volvulus decreases with age. In the present case series, 85.71% of neonates had midgut volvulus at the time of presentation, and the incidence was still higher in non-neonatal patients (18/19, 94.74%). Delayed diagnosis of midgut volvulus usually leads to massive intestinal ischemia and necrosis, presenting with hematemesis, hematochezia, abdominal distention, peritonitis, severe shock, and multiple organ dysfunction (4, 24, 25–28). Bile-stained vomiting, usually indicating severe gut pathology, must be taken seriously by all surgeons and general pediatricians (20).

Shalaby et al. (24) reported that 82% of patients experienced midgut volvulus during emergency surgery, and its acute presentation is less common beyond the neonatal period. In this study, the most frequently associated anomalies were the annular pancreas (26.67%), duodenal web (20%), intestinal atresia (20%), and ectopic pancreas (13.33%). Three of the 15 neonates had multiple anomalies, including Meckel's diverticulum, congenital heart disease, gastrointestinal duplication cyst, and trisomy 21, similar to the results described by Kedoin (1), Shehata (29), and Azzam et al. (31). Although coexisting intestinal malrotation and enteric duplication cyst or cardiac anomalies are rare, children with gut duplication or congenital heart problems should be aware of accompanying congenital malrotation (30, 31).

Although a diagnosis of malrotation should always be considered in children with bile-stained vomiting at any age (20, 26, 32), more than 10% of neonates in our study presented with symptoms of non-bilious vomiting. Similarly, bilious vomiting occurred in only 63.16% of non-neonatal patients, and other symptoms included diarrhea, small intestinal obstruction, malnutrition, and failure to thrive, making the diagnosis of malrotation more difficult (2, 14, 22, 28, 32). These findings were similar to those of a previous meta-analysis (23).

UGIs (28, 33, 34) can help make an early accurate diagnosis in different age groups. UGI examination is associated with a sensitivity of 54%–86.5% for detecting midgut volvulus (24, 27, 34). In our case series, the sensitivities of UGIs were 84.21%−87.5%. However, some subtle abnormalities and normal variants can be misinterpreted as normal or mistaken for malrotation.

US can be a useful screening and diagnostic tool (2, 24, 35–40). Abdominal US should be performed in patients with normal duodenojejunal junction on the UGIs and persistent bilious emesis (36). In our case series, the sensitivity of diagnostic US was 97.3% and 100% in the neonatal and non-neonatal groups, respectively. If a midgut volvulus exists, abdominal US can detect “whirlpool” signs, mesenteric edema, ascites, and SMV dilation (23). If the US examination is non-diagnostic and the patient is in a non-acute condition, it is vital to repeat a contrast meal to obtain optional information to diagnose malrotation (20, 40). Moreover, experienced pediatric surgeons and radiologists should play an important role in reviewing equivocal UGIs to make decisions regarding further patient care and avoid significant delays in diagnosis.

In terms of the treatment of malrotation, Ladd's procedure is usually curative (12, 24, 41). The principles of Ladd's procedure for malrotation at any age have remained unchanged with good outcomes (24, 42). However, in older children, intermittent chronic obstruction may lead to a higher emergency reoperation rate. Ladd's procedure with duodenojejunal bypass should be considered (12).

In our case series, approximately half of the non-neonatal patients received a laparoscopic or laparoscopic conversion to open Ladd's procedure, while nearly 90% of neonatal patients underwent open surgery. Laparoscopic conversion to open Ladd's procedure required a significantly longer operative time than open or laparoscopic surgery. Our study revealed that patients, especially small babies, with a high risk of conversion to an open Ladd's procedure should be identified preoperatively using the risk analysis method in future studies (5, 41). Zhu et al. (16) reported that the laparoscopic procedure for neonates with malrotation was associated with a higher risk of recurrent volvulus than open Ladd's procedure.

In terms of the management principles of patients with acute midgut volvulus and hemodynamic instability, aggressive intravenous fluid resuscitation and urgent exploratory laparotomy should be the first choices (5, 24). In terms of reoperation, the causes for reoperation were early postoperative recurrent bowel volvulus, adhesive intestinal obstruction, missed diagnosis of coexisting annular pancreas, internal hernia, and congenital duodenal web (12, 43).

This study has several limitations. The data were collected from six medical centers to improve the sample size to some extent. The total duration of the analyses was 12 years, during which time different clinical management and outcomes were inevitably achieved due to the constant updating of clinical guidelines and imaging techniques; thus, a partial risk of bias cannot be ruled out. The homogeneity of the multicenter retrospective study will be considered in future studies. Regarding the time blocks in patient management, several variables included time and location of initial presentation, US and UGI studies, and surgical consultation. Sabac et al. (26) reported that the median time from initial physician assessment and surgical consultation to surgical incision was over 14 h. The present study did not analyze the effects of these variables on the time to diagnosis. Predicting which patients will develop massive intestinal necrosis is difficult (44), multicenter clinical trials are needed to further assess how long a patient could stand volvulus and help create optimal strategies for malrotation (26).

In conclusion, malrotation with or without midgut volvulus is not rare and should be considered across all age groups. More than 10% of neonatal and nearly 40% of non-neonatal patients with malrotation present with non-bilious vomiting as the initial symptom. Diagnostic suspicion and interdisciplinary coordination are essential for the timely recognition and surgical treatment. Artificial intelligence and machine learning as a novel technique will be helpful in the detection and treatment of malrotation with volvulus (45, 46).

## Data Availability

The raw data supporting the conclusions of this article will be made available by the authors, without undue reservation.
